# Prevalence of fever and its associated risk factors among patients hospitalised with coronavirus disease 2019 (COVID-19) at the Eastern Regional Hospital, Koforidua, Ghana

**DOI:** 10.1371/journal.pone.0296134

**Published:** 2024-02-16

**Authors:** Muhyideen Alhassan Bashir, John Koku Awoonor-Williams, Forster Amponsah-Manu

**Affiliations:** 1 Medical Department, Eastern Regional Hospital, Koforidua, Ghana; 2 Department of Health Policy, Planning and Management, School of Public Health, College of Health Sciences, University of Ghana, Legon, Accra, Ghana; 3 Surgical Department, Eastern Regional Hospital, Koforidua, Ghana; University of the Witwatersrand, SOUTH AFRICA

## Abstract

**Background:**

In Ghana, temperature check at various points of entry was adopted as a means of screening people for coronavirus disease 2019 without taking into consideration data on the local prevalence of fever associated with the disease. Our objective was to assess fever prevalence and its associated risk factors among patients hospitalised with coronavirus disease 2019 at the Eastern Regional Hospital, Koforidua in Ghana.

**Methods:**

We reviewed medical records of 301 coronavirus disease 2019 patients who were admitted at the Eastern Regional Hospital, Koforidua between May 5, 2020, and August 31, 2021. Data collected on a pre-designed extraction sheet was processed, entered and analysed using Microsoft excel 2019 and Stata/IC version 16.1 software. Prevalence of fever was estimated and a multivariable logistic regression model was fitted to establish risk factors associated with fever among hospitalised coronavirus disease 2019 patients. A relationship was accepted to be significant at 5% level of significance.

**Results:**

The prevalence of fever among hospitalised coronavirus disease 2019 patients was 21.6% (95% CI, 17.1%-26.7%). Risk factors associated with fever were age group [0–19 years (AOR, 5.75; 95% CI, 1.46–22.68; p = 0.013); 20–39 years (AOR, 3.22; 95% CI, 1.42–7.29; p = 0.005)], comorbidity (AOR, 2.18; 95% CI, 1.04–4.59; p = 0.040), and disease severity [moderate (AOR, 3.89; 95% CI, 1.44–10.49; p = 0.007); severe (AOR, 4.08; 95% CI, 1.36–12.21; p = 0.012); critical (AOR, 4.85; 95% CI, 1.03–22.85; p = 0.046)].

**Conclusions:**

The prevalence of fever was low among hospitalised coronavirus disease 2019 patients at the Eastern Regional Hospital, Koforidua. However, there was an increasing risk of fever as the disease severity progresses. Fever screening may be utilised better in disease of higher severity; it should not be used alone especially in mild disease.

## 1 Introduction

Diverse healthcare systems across the globe were challenged by the coronavirus disease of 2019 (COVID-19) pandemic [1]. On the 31st December, 2019, cases of pneumonia of unknown aetiology were detected in Wuhan City, Hubei Province of China [2]. Upon investigations, the causative agent was identified as a novel coronavirus (2019-nCoV) on the 7th January 2020 [3]. Due to similarity in its genetic sequence to severe acute respiratory syndrome coronavirus (SARS-CoV), the 2019-nCoV was later named severe acute respiratory syndrome coronavirus 2 (SARS-CoV-2) while the disease associated with it was referred to as COVID-19 [3].

The World Health Organisation (WHO) on March 11, 2020 declared COVID-19 a pandemic [3]. As of 25th November 2021, there had been over 258 million reported cases of COVID-19, including over 5million deaths globally [4]. Approximately, 7.7 billion doses of COVID-19 vaccines had been served across the globe [4]. In Ghana, there had been 130,920 reported cases and 1,209 deaths as of 20th November, 2021 [5]. A total of 3,493,688 doses of COVID-19 vaccines had been administered in Ghana [5].

SARS-CoV-2 belongs to a group of viruses called coronaviruses, and it is transmitted mainly through exposure to respiratory droplets from an infected person [3]. The incubation period is 2 to 14 days after exposure [3]. Majority of COVID-19 infected individuals show no or mild symptoms, while others may present with symptoms of either moderate, severe or critical disease [3]. The symptoms include but are not limited to fever, cough, shortness of breath, body aches, fatigue, sore throat and loss of smell or taste. The disease can be complicated by sepsis, septic shock, acute respiratory distress syndrome, acute kidney injury and multi-organ failure, among others.

For the purpose of triaging and case management, the Ghana Health Service (GHS) adopted the WHO classification of COVID-19 disease severity as defined below [3].

1. *Mild Disease*: Individuals who are asymptomatic or symptomatic with non-specific symptoms such as fever, cough, sore throat, diarrhoea, headache, muscle ache, loss of smell or taste, and maintain normal oxygen saturation on room air (SpO2 ≥95%).
2. *Moderate Disease*: Patients who present with clinical evidence of pneumonia but maintain oxygen saturation of >93% on room air.
3. *Severe Disease*: Patients who present with respiratory rate of >30 cycles per minute, in respiratory distress or oxygen saturation of ≤93% on room air.
4. *Critical Disease*: Patients who present with clinical evidence of one or more of sepsis, septic shock, acute respiratory distress syndrome, respiratory failure and/or multiple organ dysfunction.

The WHO had released various protocols to guide the global emergency response to the COVID-19 pandemic. Notable among these was the application of body temperature (BT) screening to guide early detection of COVID-19 [6]. Fever or BT screening as the means to detecting COVID-19 was proposed for implementation based on the findings from the earliest study conducted in Wuhan, China, where up to 98.6% of hospitalised COVID-19 patients presented with fever [7]. In Ghana, it was a requirement to provide thermometer guns and use same to check BT at various entry points and gatherings, as a way of detecting COVID-19 cases early in order to intervene [8]. However, the novel coronavirus had been unpredictable with its epidemiological and clinical features [6].

Fever usually occurs when one’s BT rises above the person’s known normal daily values, primarily in response to an infectious agent [9, 10]. In practice, fever refers to an elevated BT >37.4˚C when a contact thermometer is placed in a person’s armpit [11]. For the sake of infection control, a noncontact thermometer is preferred when screening patients for COVID-19, despite no consensus on the cut-off temperature for this device [12].

Since the onset of COVID-19 pandemic emphasis has been on temperature screening at various points of entry as a way of early detection of cases [6]. In a systematic review and meta-analysis involving published data worldwide, the prevalence of fever among adult COVID-19 patients was as high as 79.43% [13]. Our search did not identify any data in literature on the prevalence of fever among COVID-19 patients in sub-Saharan Africa. However, two different studies conducted on the clinical characteristics of COVID-19 in Accra, Ghana showed a low prevalence of fever [14, 15]. Ashinyo et al. reported 29.6% fever prevalence in a retrospective study of some discharged COVID-19 patients [14], whereas Oduro-Mensah et al. reported 7.6% of patients who had a history of fever as a symptom of COVID-19 [15].

Evidence was therefore scanty if BT screening was the most appropriate or rapid screening tool for detecting COVID-19 in a sub-Saharan African country like Ghana. In the Eastern Regional Hospital, Koforidua (ERHK), we observed that a number of symptomatic clients visiting the hospital pass BT screening, only for them to be diagnosed of COVID-19 on subsequent assessment by physicians. Yet there had been increasing demand for logistics and human resource by healthcare workers to man and check BT at COVID-19 screening centres and various points of entry across the country [3].

On the other hand, there were some reports of fever or peak BT being associated with mortality among COVID-19 patients [16]. Unremitting high-grade fever can be counterproductive during a dysregulated inflammatory process described as cytokine storm [17]. This was evidenced in a study conducted in an intensive care unit (ICU) setting where there was a linear relationship between BT and mortality [16]. While overall mortality was 61.1% for the studied population, mortality was lower (40%) among patients with peak temperature less than 38.9˚C [16]. Conversely, mortality was higher (70.6%) among patients with peak temperature greater than 40˚C and there was 100% mortality among the 14 patients who had hyperthermia greater than 40.6˚C [16]. In another study conducted in the United States it was reported that maximum BT during COVID-19 infection was significantly correlated with mortality rate [18]. For every 0.5°C increase in BT there was a significant increase in mortality; the mortality was as high as 42% in those with maximum BT > 40.0°C [18].

In view of the above data on fever and mortality, and the inconsistent data on fever prevalence among COVID-19 patients, our study sought to estimate the prevalence of fever and unravel the risk factors associated with fever among symptomatic COVID-19 patients. Thus, the objective of this study was to assess prevalence of fever and its associated risk factors among patients hospitalised with COVID-19 at the ERHK between 5^th^ May, 2020 and 31^st^ August, 2021.

## 2 Methods

### 2.1. Study design

We conducted a cross-sectional retrospective study to assess the prevalence of fever and its associated risk factors among patients hospitalised with COVID-19 at the ERHK between May 5, 2020, and August 31, 2021. Electronic medical records of eligible participants who were discharged during the period were reviewed. Data were collected using a predesigned extraction sheet. Data was then processed, analysed and results presented without any further follow-up [19].

### 2.2. Setting

The study was carried out at the ERHK, located in the administrative capital of the eastern region of Ghana. The region is found in the south eastern part of Ghana and has a total land area of 19, 323 square kilometres, constituting 8.1% of Ghana’s total land area [20].

The region has a population of 2,925,653 making it the 3^rd^ most populous in Ghana after Greater Accra Region and Ashanti Region [21]. The population is 50.9% female and 49.1% male with a Rural-Urban split of 54.1% and 45.9% respectively [21].

The region has a total of 1,226 healthcare facilities under 33 Municipal/ District Health Directorates for which ERHK serves as the main referral facility [22]. Its catchment area includes adjoining districts in the Central, Greater Accra, Ashanti, Bono East and Volta regions.

The hospital operates under the GHS and provides comprehensive primary and specialist care in all major aspects of health care. The services include Obstetrics and Gynaecology, Internal Medicine, Paediatrics, General Surgery, Dental care, and Ophthalmology, among others.

The medical department manages the Isolation and Treatment Centre in collaboration with the Institutional Public Health Unit. The Isolation and Treatment Centre has a 13-bed capacity ward designated at the onset of COVID-19 pandemic for inpatient management of the disease. The hospital had the laboratory capacity of testing for COVID-19. During the period of the pandemic there had been evolving case definitions which GHS had adopted to guide diagnosis. Individuals who fitted into the suspected case definition were tested with polymerase chain reaction (PCR) performed on specimens taken from nasopharyngeal swab. The hospital recorded its first case of COVID-19 on the 5th May, 2020.

At the beginning, all persons who tested positive were routinely admitted until the year 2021 when a guideline for home isolation was instituted for patients with mild disease. However, majority of patients with COVID-19 irrespective of the severity were admitted on account that their residential status did not qualify for home-isolation. There was a functional COVID-19 management team comprising diverse health workers including physicians, nurses, disease control officers, pharmacists, anaesthetists, medical laboratory scientists, among others who were specially trained to provide both clinical and public health care to the COVID-19 patients.

Though a single centre, the ERHK was chosen for this study because the region found itself between the two most populous regions in Ghana (i.e., Greater Accra and Ashanti), and as such, “influx” of COVID-19 cases from the neighbouring regions was expected.

### 2.3. Study participants

The study target was persons of all age group who were diagnosed with COVID-19 through a PCR confirmation, and were admitted to the COVID-19 Isolation and Treatment Centre of the ERHK either from home through triage or from any of the traditional wards of the hospital.

#### Inclusion criteria

Folders of all confirmed COVID-19 patients who were hospitalised and discharged at the ERHK between May 5, 2020, and August 31, 2021 were eligible to be included in the study. This was necessary at the time because the first case of COVID-19 which required admission at the ERHK was recorded on the 5th May, 2020 while data extraction was done in September, 2021.

#### Exclusion criteria

Folders of patients with no records of temperature at presentation and patients with clinically invalid temperature were excluded from the study. This was applied because ‘fever’, as the primary dependent variable of the study was only derived from the level of BT at presentation.

### 2.4. Sampling method

All the 307 records available at the time of data collection were reviewed without further sampling. At the end of the review, 301 patients were finally enrolled into the study for analysis after applying the exclusion criteria.

### 2.5. Data collection

A pre-designed data extraction sheet in a Microsoft excel file was used to compile relevant information during records review of hospitalised COVID-19 patients who were discharged between 5^th^ May, 2020, and 31^st^ August, 2021. Data collection was undertaken solely by the authors between 21^st^ and 29^th^ September, 2021. The folder numbers of the participants were obtained from the admission book at the Isolation and Treatment Centre of the ERHK while records of participants were accessed from the electronic management system using their folder numbers. Raw data on socio-demographics, epidemiology, comorbidity, body temperature, oxygen saturation, disease severity and discharge outcome were extracted. Data was collected anonymously. Information that could identify individual participants including names were not reviewed or extracted during and after data collection.

#### 2.5.1. The data extraction sheet and study variables

*Socio-demographic and epidemiological characteristics*. In this section, data on patient’s sex, age in completed years, date of admission and date of discharge were collected. Variable named ‘sex’ was collected on a nominal scale and binary levels as ‘male’ or female. ‘Age’ though a continuous variable, was collected in discrete form (i.e., completed years of life) measured on a ratio scale. This was done to allow appropriate age categorisation during data entry. Both the date of admission and discharge were collected in the format ‘dd/mm/yyyy’, which helped us derive the year of COVID-19 infection.

*Comorbidity*. This section of the data collection helped us to extract list of comorbidities recorded for each participant. This included concurrent infection(s), pre-existing or chronic diseases identified by physicians during case management.

*Temperature at presentation*. Temperature measured in degree Celsius, which was first recorded at presentation of patient was collected as a continuous variable on an interval scale. For cases detected from the hospital’s triage area the temperatures recorded during triaging were collected. However, there was a category of patients who were already on admission in the traditional wards of the hospital prior to the detection of COVID-19. For such patients the temperature values recorded at the first clinical suspicion were the ones collected for the purpose of our study.

*Prevalence of fever*. Fever, which was operationally defined as an elevated temperature of >37.4˚C at the time of presentation was derived from the recorded temperature. Patient’s status on fever was then collected as a binary outcome variable with levels ‘fever’ or ‘no fever’.

*Severity of disease at presentation*. Data collected under this section were patients’ oxygen saturation and disease severity at presentation. Oxygen saturation in percentage (%) was a continuous variable measured on a ratio scale. The variable ‘disease severity’, which was measured on an ordinal scale based on standardised clinical features at the time of triaging by physicians was also collected [3]. The ordinal levels assigned to the variable ‘disease severity’ were ‘mild’, ‘moderate’, ‘severe’ and ‘critical’.

*Discharge outcome (mortality)*. The variable named ‘discharge outcome (mortality)’ was a binary data with levels ‘discharged alive’ and ‘discharged dead’ to signify the presence and absence of mortality respectively. This data was collected from the software interphase named ‘discharge outcome’ on the electronic management system.

#### 2.5.2. Quality assurance

In order to address potential sources of bias, we extracted all data in the best possible manner. Few scientifically invalid data were detected on the electronic medical records. However, triage sheets for such patients were used to cross-validate data. The extraction sheet was thoroughly examined for completeness and errors.

The completed data extraction sheet was protected with a password and copies saved on a personal computer.

### 2.6. Data entry and processing

Each row, representing data of a participant on the extraction sheet was assigned a serial number. New variables such as ‘age-group’, ‘year of infection’, ‘comorbidity’, and ‘fever’ were derived from the raw data for the purpose of data entry and analysis. Age was categorised as 0–19 years, 20–39 years, 40–59 years and ≥60 years. The date of admission and date of discharge were used to derive the ‘year of infection’ (signifying the calendar year patient got infected). Comorbidity was categorised as having one or more comorbidity versus none. A body temperature >37.4˚C was considered as having fever [11]. Categorical data were then coded appropriately and entry done into another excel sheet using Microsoft Excel 2019.

Data entry file was converted into a comma-separated values (CSV) file before importing into STATA statistical software for analysis.

### 2.7. Statistical analysis

Descriptive analysis was first conducted to summarise baseline characteristics of hospitalised COVID-19 patients at the ERHK. For categorical data including sex, age-group, comorbidity, year of infection, disease severity, discharge outcome, and fever, a frequency table was generated. Frequencies and percentages for these categorical variables were then presented. For each continuous variable including age, temperature, and oxygen saturation, test for normality was conducted using both histogram plot and Shapiro-Wilk test. All the three (3) continuous variables were not normally distributed. They were therefore summarised and reported as median age, median temperature, and median oxygen saturation together with their respective range and interquartile range.

To estimate the prevalence of fever with 95% confidence interval among hospitalised COVID-19 patients at the ERHK, a proportion estimate using fever as an outcome variable was computed and output was generated on the software.

Given that the variable ‘fever’ was a binary outcome, and there were multiple predictor variables considered in our study, the risk factors associated with fever among hospitalised COVID-19 patients were determined using Multivariable Binomial Logistic Regression. The model was fitted in a forward-stepwise approach using sex, age-group, comorbidity, disease severity, discharge outcome, and oxygen saturation to predict the occurrence of fever among hospitalised COVID-19 patients. The model fitted the data well (LR X^2^ (10), 24.6; p-value, 0.006). Analysis of the Receiver-Operating Characteristics (ROC) was computed to assess the performance of the model in predicting the occurrence of fever relative to others. The model performed well (Area under ROC curve, 0.71). Adjusted odds ratios (AOR) together with their respective 95% confidence intervals and p-values for predictors were summarised in a tabular form. Variables with p-value <0.05 were considered as significantly associated with fever among hospitalised COVID-19 patients.

All results were presented mainly in texts and tables. All analyses were performed at 5% level of significance using Stata/IC version 16.1 software.

### 2.8. Ethical approval and consent to participate

Ethical clearance for the study was obtained from the Ethics Review Committee of Ghana Health Service with reference number: GHS-ERC045/08/21. Also, a written permission was obtained from the management of the ERHK to access patients’ medical records for the purpose of the study. Data were collected solely from existing medical records and analysed anonymously. At the time of data extraction, the participants were not available in the hospital and hence informed consent could not be obtained. The need for consent was waived by the ethics committee during the review process.

## 3 Results

### 3.1. Baseline characteristics of hospitalised COVID-19 patients

A total of 307 folders of patients admitted and discharged at the ERHK were identified. Out of this number, there were missing records of temperatures for 6 patients and therefore were excluded from the analysis.

Table 1 presents the demographic, epidemiological and clinical characteristics of COVID-19 patients at the ERHK. Out of 301 patients who were included in the final analysis, 148 (49.2%) were males and 153 (50.8%) were females. The median age of patients was 56 years (IQR, 36–70 years; range, 0.3–102 years). As many as 45.5% of the patients were ≥60 years old, while only 4.6% were between the age of 0–19 years old. There was a surge in the total number of cases in the year 2021 (during the first 8 months) compared to 2020 (232, 77.1% versus 69, 22.9%). One or more comorbidities were identified in 210 (69.8%) patients. Patients presented with a median temperature of 36.6°C (IQR, 36.3–37.2°C; range, 34.7–40.0°C), and 21.6% (65/301) of these patients had fever. The median oxygen saturation at presentation was 91% (IQR, 84–98%; range, 27–100%). Half (151/301) of the COVID-19 patients presented with severe disease; 7.3% (22/301) presented with critical disease. Sixty-seven out of the 301 individuals (22.3%) were discharged dead following hospitalisation.

**Table 1:** Baseline characteristics of hospitalised COVID-19 patients.

	No. (%)
Variable	N = 301
**Demographic and epidemiological data**	
Age, median (IQR) [range], years	56 (36–70) [0.3–102]
Age-group	
0–19 years	14 (4.6)
20–39 years	77 (25.6)
40–59 years	73 (24.3)
≥ 60 years	137 (45.5)
Sex	
Male	148 (49.2)
Female	153 (50.8)
Year of infection	
2020	69 (22.9)
2021	232 (77.1)
**Clinical data**	
Presenting temperature, median (IQR) [range], ˚C	36.6 (36.3–37.2) [34.7–40.0]
Fever (Presenting temperature >37.4 ˚C)	65 (21.6)
No fever	236 (78.4)
Comorbidity	
One/more comorbidities ^a^	210 (69.8)
No comorbidity	91 (30.2)
Presenting Oxygen saturation, median (IQR) [range], %	91 (84–98) [27–100]
Disease severity **^b^**	
Mild	66 (21.9)
Moderate	62 (20.6)
Severe	151 (50.2)
Critical	22 (7.3)
Discharge outcome	
Discharged alive	234 (77.7)
Discharged dead	67 (22.3)

Abbreviations and symbols: COVID-19, coronavirus disease 2019; IQR, interquartile range; No., number of observation or frequency; N, total sample size.

^a^Comorbidities identified among others were hypertension, diabetes, heart failure, asthma, retroviral infection, pulmonary tuberculosis, lung fibrosis, chronic kidney disease, and sickle cell disease.

^b^Disease severity was determined using presenting oxygen saturation, clinical symptom(s) of pneumonia and the presence or absence of end organ damage [3].

Out of the 65 patients who presented with fever, 8 (12.3%) had mild disease, 19 (29.2%) had moderate disease, 32 (49.2%) had severe disease, and 6 (9.2%) had critical disease.

### 3.2. Prevalence of fever among hospitalised COVID-19 patients

We found that the prevalence of fever was 21.6% (95% CI, 17.1%-26.7%; SE, 2.4%) among all hospitalised COVID-19 patients.

Table 2 depicts the prevalence of fever in different subgroups of COVID-19 patients. There was equal fever prevalence among male (prevalence, 21.6%; 95% CI, 15.3%-29.1%) and female (prevalence, 21.6%; 95% CI, 15.3%-28.9%) COVID-19 patients. Individuals between the age of 0–19 years had a fever prevalence of 35.7% (95% CI: 12.8%-64.9%) while elderly persons (≥60 years) had fever prevalence of 17.5% (95% CI: 11.6%-24.9%). Patients who presented with one/more comorbidities had fever prevalence of 24.8% (95% CI: 19.1%-31.2%) while those without comorbidity had fever prevalence of 14.3% (95% CI: 7.8%-23.2%). The prevalence of fever in the different severity groups were 12.1% (95% CI: 5.4%-22.5%) for mild disease, 30.6% (95% CI: 19.6%-43.7%) for moderate disease, 21.2% (95% CI: 15.0%-28.6%) for severe disease, and 27.3% (95% CI: 10.7%-50.2%) for critical disease. The prevalence of fever among COVID-19 patients who were discharged dead was 28.4% (95% CI: 18.0%-40.7%) as against 19.7% (95% CI: 14.8%-25.3%) in those who were alive.

**Table 2:** Prevalence of fever in different subgroups of COVID-19 patients.

Subgroup	Number of patients	Fever prevalence [95% CI] (%)
**Sex**		
Male	148	21.6 (15.3–29.1)
Female	153	21.6 (15.3–28.9)
**Age group**		
0–19 years	14	35.7 (12.8–64.9)
20–39 years	77	24.7 (15.6–35.8)
40–59 years	73	23.3 (14.2–34.6)
≥ 60 years	137	17.5 (11.6–24.9)
**Year of infection**		
2020	69	15.9 (8.2–26.7)
2021	232	23.3 (18.0–29.3)
**Comorbidity**		
No comorbidity	91	14.3 (7.8–23.2)
One/more comorbidities^a^	210	24.8 (19.1–31.2)
**Disease severity ^b^**		
Mild	66	12.1 (5.4–22.5)
Moderate	62	30.6 (19.6–43.7)
Severe	151	21.2 (15.0–28.6)
Critical	22	27.3 (10.7–50.2)
**Discharge outcome**		
Alive	234	19.7 (14.8–25.3)
Dead	67	28.4 (18.0–40.7)

Abbreviations and symbols: COVID-19, coronavirus disease 2019; CI, confidence interval.

**^a^**Comorbidities identified among others were hypertension, diabetes, heart failure, asthma, retroviral infection, pulmonary tuberculosis, lung fibrosis, chronic kidney disease, and sickle cell disease.

**^b^**Disease severity was determined using presenting oxygen saturation, clinical symptom(s) of pneumonia and the presence or absence of end organ damage [3].

### 3.3. Risk factors associated with fever among hospitalised COVID-19 patients

The risk factors associated with fever were analysed with logistic regression model (LR X^2^ (10), 24.60; p-value, 0.006; area under ROC curve, 0.71), controlling the effects of sex, age group, comorbidity, diseases severity, discharge outcome, and oxygen saturation.

As portrayed in Table 3, odds of fever did not significantly differ in female COVID-19 patients relative to males (AOR, 1.12; 95% CI, 0.62–2.00; p = 0.710). However, we found that patients from the age of 0 to 19 years (AOR, 5.75; 95% CI, 1.46–22.68; p = 0.013), and 20 to 39 years (AOR, 3.22; 95% CI, 1.42–7.29; p = 0.005) were significantly associated with higher odds of fever when compared to patients who were 60 years old and above. There was no statistically significant difference in the odds of fever in patients who were 40 to 59 years old compared to 60 years old and above (AOR, 1.67; 95% CI, 0.80–3.50; p = 0.171). There was over 2-fold higher odds of fever among patients who had one or more comorbidities (AOR, 2.18; 95% CI, 1.04–4.59; p = 0.040) compared to patients who had no comorbidity. Moderate disease, severe disease and critical disease were associated with 3.89-folds (95% CI, 1.44–10.49; p = 0.007), 4.08-folds (95% CI, 1.36–12.21; p = 0.012), and 4.85-folds (95% CI, 1.03–22.85; p = 0.046) higher odds of fever respectively, compared to mild disease. The odds of fever among patients who were discharged dead versus alive was statistically not significant (AOR, 1.86; 95% CI, 0.89–3.88; p = 0.098).

**Table 3:** Multivariable logistic regression analysis of factors associated with fever among hospitalised COVID-19 patients.

Factor	Adjusted Odds Ratio	95% CI	P-value
**Sex**			
Male	1.00 (reference)		
Female	1.12	0.62–2.00	0.710
**Age group**			
0–19 years	5.75	1.46–22.68	**0.013**
20–39 years	3.22	1.42–7.29	**0.005**
40–59 years	1.67	0.80–3.50	0.171
≥ 60 years	1.00 (reference)		
**Comorbidity**			
No comorbidity	1.00 (reference)		
One/more comorbidities ^a^	2.18	1.04–4.59	**0.040**
**Disease severity ^b^**			
Mild	1.00 (reference)		
Moderate	3.89	1.44–10.49	**0.007**
Severe	4.08	1.36–12.21	**0.012**
Critical	4.85	1.03–22.85	**0.046**
**Discharge outcome**			
Alive	1.00 (reference)		
Dead	1.86	0.89–3.88	0.098
**Presenting-oxygen saturation, %**	1.03	0.99–1.06	0.116

Abbreviation: CI, confidence interval.

**^a^**Comorbidities identified among others were hypertension, diabetes, heart failure, asthma, retroviral infection, pulmonary tuberculosis, lung fibrosis, chronic kidney disease, and sickle cell disease.

**^b^**Disease severity was determined using presenting oxygen saturation, clinical symptom(s) of pneumonia and the presence or absence of end organ damage [3].

P-values highlighted in bold were considered statistically significant (p<0.05).

## 4 Discussion

### 4.1. Characteristics of hospitalised COVID-19 patients

Over the 16-month period, a total of 307 patients were admitted and discharged at the Isolation and Treatment Centre of the regional hospital in Koforidua. This figure was coincidentally the same as the number recorded at the two centres in the Greater Accra Region between the months of March and June of the year 2020 [14].

In our study, there were slightly more females (50.8%) than males (49.2%) contrary to reports from previous studies conducted in Accra, where the prevalence of COVID-19 was higher among males [14, 15]. Our findings on sex distribution were as well not consistent with what had been reported in a number of studies conducted outside Ghana, including what was reported in a New York City hospital [23].

The average age of participants was 56 years as against 37.9 years and 40.7 years respectively found in the two different studies conducted in Accra [14, 15]. Even though only 4.6% of patients were between the age of 0–19 years, cumulatively, patients less than 60 years old were the majority.

There was a surge in the COVID-19 admitted cases at the ERHK in the first 8 months of 2021, which contributed up to 77.1% of the cases admitted within the first 16 months of COVID-19 admissions in the hospital. This rise may be attributed to a possible increased viral spread among people during the Christmas, New year, Easter and Eid festivities which happened consecutively from December, 2020.

One or more comorbid conditions including hypertension, diabetes, heart failure, asthma, retroviral infection, pulmonary tuberculosis, lung fibrosis, chronic kidney disease, and sickle cell disease were identified in over 69% of patients in our study, which was far higher than 25.1% and 39% reported in Accra [14, 15]. This may be attributed to the nosocomial COVID-19 infection among in-patients who were otherwise admitted on account of pre-existing conditions prior to their translocation to the Isolation and Treatment Centre at the ERHK.

Our study found a median temperature at presentation of 36.6˚C, which is clinically consistent with the mean temperature of 36.3˚C reported in the previous study in Accra [14]. The significance here was that, on average COVID-19 patients were presenting with temperatures less than the cut off value for fever. Tharakan and his colleagues also reported an average BT of 37.0˚C at first encounter in Mount Sinai [18]. This consistent normal average temperature indicates that the viral illness on average does not cause elevated body temperature.

Up to 50% of hospitalised patients presented with severe disease in our study. As low as only 5.1% presented with moderate to severe disease in the study conducted by Oduro-Mensah et al., which analysed both outpatient and in-patient data [15]. This discrepancy was widened by the increased cases of nosocomial COVID-19 among hospitalised patients who already were severely ill at the ERHK before testing positive to SARS-CoV-2. It may as well explain the high percentage (22.3%) of patients who were discharged dead in our study.

### 4.2. Prevalence of fever among COVID-19 patients

Contrary to the report of 98.6% fever prevalence among hospitalised COVID-19 patients in one of the earliest studies conducted in Wuhan, China [7], and that of 79.43% fever prevalence from the recent systematic review and meta-analysis [13], the prevalence of fever in our study was as low as 21.6% (95% CI, 17.1%-26.7%). There was 95% confidence that the true prevalence of fever will fall between 17.1% and 26.7%. Our finding is only slightly lower than 29.6% reported in Accra [14]. Therefore, a more significant number of COVID-19 cases may be missed by the sole reliance on fever or temperature screening in Ghana.

Even though the prevalence of fever in our study is generally low, patients between the age of 0 and 19 years presented with relatively higher prevalence of fever than the elderly (≥ 60 years old) patients (35.7% vs 17.5%). This pattern is inconsistent with findings from the recent meta-analysis where the fever prevalence among paediatric subjects was relatively lower compared to adult subjects [13].

In terms of disease severity, our study estimated fever prevalence in mild disease as 12.1% (95% CI: 5.4%-22.5%); in moderate disease as 30.6% (95% CI: 19.6%-43.7%); in severe disease as 21.2% (95% CI: 15.0%-28.6%); and in critical disease as 27.3% (95% CI: 10.7%-50.2%). Unlike the disease severity classification [3] adopted in our study, the published meta-analysis classified severity broadly into severe versus non-severe disease [13], making it difficult to compare findings. Nevertheless, the lowest prevalence of fever in individuals with mild disease (12.1%) in our study suggests poor utility of fever screening in early detection of COVID-19.

### 4.3. Risk factors associated with fever among hospitalised COVID-19 patients

We assessed risk factors associated with fever after estimating the fever prevalence among hospitalised COVID-19 patients at the ERHK. To the best of our knowledge, literature on the risk factors associated with fever in COVID-19 in sub-Saharan Africa is scarce. From our study, the risk factors significantly associated with fever among hospitalised COVID-19 patients were found to be young age group (0–19 years and 20–39 years), presence of one or more comorbidities, and higher disease severity.

Young COVID-19 patients who were either between 0 and 19 years or 20 and 39 years had higher odds of fever relative to the older patients. That means young COVID-19 patients were more likely to present with fever. These individuals tend to mount better febrile response to infection [24]. Consequently, temperature screening, despite its limitations was likely to detect COVID-19 better among the younger population compared to the older ones.

The second risk factor found to be associated with fever was comorbidity. Patients with one or more comorbidities compared to those without any comorbidity had more than a 2-fold odds of presenting with fever. Despite lack of comparative data, the association between comorbidity and fever was likely to be multifactorial. It may be argued that some other fever-causing disease conditions among the patients with at least one comorbidity might had independently led to fever. Our study did not analyse how each comorbidity affected the presence of fever among the subjects. Nevertheless, comorbidity in general played a role in the prevalence of fever among hospitalised COVID-19 patients. That means temperature screening may detect COVID-19 patients better among those with at least a comorbidity. More so, such group of patients may require close monitoring and management, considering the literature that supports the link between fever and mortality [18].

We found that different levels of disease severity were associated with the odds of fever. The higher the level of disease severity, the greater the odds of fever. This finding is consistent with the assumption that disease severity increases the body’s inflammatory processes and therefore may increase body temperature. The association found between severity of disease and fever also conforms with findings in previous studies [25, 26]. The implication of this finding is that temperature screening may detect more COVID-19 cases at late stage of the disease. Using temperature screening alone therefore may be misleading in early detection of patients with the disease.

The biological sex of patients did not show any significant association with fever among hospitalised COVID-19 subjects, despite immunity and hormonal differences between male and female known in literature [27, 28]. Additionally, discharge outcome or death was not significantly associated with fever in our current study, unlike what was reported by Tharakan and his colleagues [18]. In the Ghanaian setting, fever may not necessarily be useful in mortality risk assessment, though higher level of study is required to cross-validate this finding.

### 4.4. Study limitations

Our study has limitations. The study was limited to only one COVID-19 Isolation and Treatment Centre in a single region among 16 regions in Ghana. Findings therefore may be difficult to be generalised to the larger population of Ghana. Also, records on socio-demographic and epidemiological data were limited to only sex, age and year of infection for majority of eligible participants on the electronic medical system of the hospital. Factors such as body mass index, place of residence, occupation, travel history, among others were therefore not included in the data collection and analysis. The COVID-19 pandemic itself placed some restrictions on us as researchers in the design and conduct of this study.

### 4.5. Validity and reliability

Our study was designed with the validity and reliability in mind. The COVID-19 patients who were included in the study were persons of all age group, sex, and social class; residing within and outside the borders of the Eastern Region of Ghana. As well, the hospital had the capacity to manage COVID-19 patients of any severity. Participants cut across all levels of severity the disease could manifest. Moreover, the data collected were from the cases managed spanning from the onset of COVID-19 pandemic through period of emergence of new viral strains to the full enrolment of COVID-19 vaccines in the region. The temperatures recorded of patients in the hospital during the pandemic were done with the handheld infrared thermometer which was acceptable globally. However, the temperature values recorded for the category of patients who were on admission prior to the diagnosis of COVID-19 may not be entirely valid as the management of index disease admission might have influenced the temperature at the time of suspicion. Although the oxygen saturations that contributed to the disease severity classification of patients were measured with pulse oximeters of diverse brands, the devices were regularly serviced by the Quality Assurance Unit of the hospital.

The external validity however could not be assured since the study was conducted in a single centre/region. The study participants may not be representative of all COVID-19 patients. This limits the generalisability of the findings to other settings. However, the findings could be generalisable to similar settings and might have at least generated hypothesis that are worth testing in future studies.

## 5 Conclusions

Our study estimated the prevalence of fever among patients hospitalised with COVID-19 as 21.6%. Thus, a more significant number of COVID-19 cases were likely being missed with the temperature screening policy in Ghana [6]. Risk of fever increases, as the COVID-19 disease severity progresses. Therefore, temperature screening may yield a better utility when applied on individuals who present with high disease severity than its sole usage in the general population. The temperature screening may not be a rapid tool or enough for detecting individuals with COVID-19 especially in mild/asymptomatic disease. A checklist of other symptoms/signs should be employed in conjunction with the temperature screening to help improve early case detection. A higher-level study should however be conducted to cross-validate and enhance the generalisability of this research findings beyond the ERHK.

## Supporting information

S1 File Data extraction sheet. ˚C, degree Celsius; Rows of data highlighted in gold colour have missing temperatures and were excluded in the final analysis.
(XLSX)

S2 File Completed STROBE checklist. (DOCX)

S3 File Dataset. (CSV)

S4 File Full results of multivariable logistic regression model for risk factors associated with fever among hospitalised COVID-19 patients. (DOCX)
