# Supplementary material for: Prevalence of fever and its associated risk factors among patients hospitalised with coronavirus disease 2019 (COVID-19) at the Eastern Regional Hospital, Koforidua, Ghana
Source: PLoS One. 2024 Feb 16;19(2):e0296134. doi: 10.1371/journal.pone.0296134 (PMC10871519; doi:10.1371/journal.pone.0296134)
Supplement: Supplementary file 3 [file pone.0296134.s004.docx]

```
. logistic fever i.sex b4.age_cats i.comorb i.pres_sev i.outcome c.spo2, base

Logistic regression                             Number of obs     =        301
                                                LR chi2(10)       =      24.60
                                                Prob > chi2       =     0.0062
Log likelihood = -144.74197                     Pseudo R2         =     0.0783
```

fever	Odds Ratio	Std. Err.	z	P>|z|	[95% Conf.	Interval]
sex						
male	1	(base)				
female	1.116937	.3317656	0.37	0.710	.6240144	1.999228
age_cats						
min-19 years	5.748203	4.026149	2.50	0.013	1.456581	22.68452
20-39 years	3.222546	1.341791	2.81	0.005	1.424882	7.28818
40-59 years	1.673163	.6289445	1.37	0.171	.8008854	3.495475
60+ years	1	(base)				
comorb						
no comorbidity	1	(base)				
atleast 1 comorbidity	2.183224	.8284604	2.06	0.040	1.037758	4.593046
pres_sev						
mild	1	(base)				
moderate	3.889762	1.968237	2.68	0.007	1.442817	10.4866
severe	4.08095	2.281851	2.52	0.012	1.364	12.20979
critical	4.851892	3.835915	2.00	0.046	1.030266	22.84929
outcome						
alive	1	(base)				
dead	1.859956	.6972969	1.66	0.098	.8920467	3.87809
spo2	1.028298	.0182352	1.57	0.116	.9931715	1.064666
_cons	.002003	.0037415	-3.33	0.001	.0000515	.07793

```
Note: _cons estimates baseline odds.

. lroc

Logistic model for fever

number of observations =      301
area under ROC curve   =   0.7088
```
